# Whether individualized dose escalation should be recommended for lymph nodes with different sizes in the definitive radiotherapy of cervical cancer?

**DOI:** 10.1186/s13014-022-02132-0

**Published:** 2022-10-20

**Authors:** Xiaojuan Lv, Huiting Rao, Tao Feng, Chufan Wu, Hanmei Lou

**Affiliations:** 1 Department of Gynecologic Radiation Oncology, The Cancer Hospital of the University of Chinese Academy of Sciences (Zhejiang Cancer Hospital), Banshan East Road 1, Hangzhou, 310022 Zhejiang China; 2 Institute of Basic Medicine and Cancer (IBMC), Chinese Academy of Sciences, Banshan East Road 1, Hangzhou, 310022 Zhejiang China; 3 The Second Affiliated College of Zhejiang Chinese Medical University, Zhejiang Chinese Medical University, Binwen Road 548, Hangzhou, 310053 Zhejiang China

**Keywords:** Radiotherapy, Dose escalation, Lymph node, Uterine cervical neoplasms

## Abstract

**Background and purpose:**

Dose escalation for positive node maybe improve the regional control of patients with node-positive cervical cancer, but the optimal dose for nodes of different sizes remains controversial. The purpose of this study was to explore the individualized dose escalation for lymph nodes (LNs) with different sizes in the definitive radiotherapy of cervical cancer.

**Methods:**

A total of 1002 cervical cancer patients with the International Federation of Gynecology and Obstetrics (FIGO 2009) stage IB1–IVA, who were treated by definitively radiotherapy between September 2013 and December 2016 were enrolled. All LNs identified by computed tomography/magnetic resonance imaging (CT/MRI) were assigned into three groups according to the short diameters of < 1 cm, 1–2 cm or ≥ 2 cm at pretreatment.

**Results:**

In total, 580 patients with 1310 LNs were detected. The nodal control rate in groups of LNs < 1 cm, 1–2 cm and ≥ 2 cm was 99.4%, 96%, and 75.9%, respectively (P = 0.000). Among LNs < 1 cm, the control, overall survival (OS) and progression-free survival (PFS) rates did not significantly differ among three dose-based groups (≤ 50.4 Gy, 50.4–60 Gy, > 60 Gy) (control rate, 99.4% vs. 99.3% vs. 100%, P = 0.647) (5-year OS, 76.2% vs. 79% vs. 81.6%, P = 0.682) (5-year PFS, 74.1% vs. 73.9% vs. 78.9% P = 0.713). Among LNs of 1–2 cm, the control and PFS rates were significantly higher in the group of dose ≥ 55 Gy than the group of dose < 55 Gy (control rate, 98% vs. 93.6%, P = 0.028) (5-year PFS, 69.6% vs. 56.7%, P = 0.025). However, this did not cause a significant difference for 5-year OS rate (72.6% vs. 68.3%, P = 0.5). Among LNs ≥ 2 cm, the control, OS, and PFS rates were higher in the group of dose ≥ 55 Gy than the group of dose < 55 Gy, while no significant difference was found (control rate, 82.1% vs. 63.2%, P = 0.107) (5-year OS, 60.6% vs. 37.5%, P = 0.141) (5-year PFS, 51.5% vs.37.5%, P = 0.232).

**Conclusions:**

Radiation dose escalation is not necessary for LNs < 1 cm, and dose escalation of 55 Gy is enough for LNs of 1–2 cm.

## Background

Lymph node (LN) status is an important prognostic factor of patients with cervical cancer [1–3]. The International Federation of Gynecology and Obstetrics (FIGO) staging system of cervical cancer (ver. 2018) has highly emphasized on LN assessment [4]. The survival rate of patients with cervical cancer and metastatic LNs has significantly decreased [5–8]. Treatment intensification may be necessary for those patients with cervical cancer, and radiation dose escalation may be an option to overcome the worse outcomes.

The development of imaging techniques and radiotherapy enables accurate delivery of radiation dose escalation to LNs of patients with cervical cancer [8, 9]. Although some studies have shown that the positive LNs could be well controlled without an additional nodal boost after whole pelvic radiotherapy of 45–50.4 Gy [10, 11], more studies recommended that nodal boost could improve the regional control of patients with node-positive cervical cancer and the toxicity was tolerated [9, 12–14]. However, the optimal dose for positive LNs still remains controversial [7, 9–15].

LN size was shown to be an important factor of survival, in which patients with bulky nodes have poorer survival outcome than those with smaller nodes, and a higher radiation dose may be essential to control bulky LNs [9, 15, 16]. While some reports have shown that patients with relatively small LNs did not benefit from dose escalation [16, 17]. Individualized dose escalation maybe essential for lymph nodes of different sizes. The aim of the present study was to evaluate the influences of radiation dose escalation on LN control and survival in definitive radiotherapy of patients with cervical cancer, and to explore the individualized dose escalation recommendation for LNs with different sizes.

## Methods

### Patients

Data of patients with cervical cancer who received definitive radiotherapy alone or concurrent chemoradiotherapy (CCRT) between September 2013 and December 2016 at Zhejiang Cancer Hospital (Hangzhou, China) were retrospectively analyzed. All medical records were analyzed after an approval from the Ethics Committee of Zhejiang Cancer Hospital.

Patients included in our analysis were those with FIGO (2009) stage IB1–IVA [18], who were treated definitively, and with follow-up visits. Patients treated with palliative intent, unfinished treatment, with special pathological type, and those without follow-up visits were excluded. Finally, a total of 1002 patients were involved in our study. Age, FIGO stage, histological type, LN status, treatment, complications, and follow-up data were recorded and analyzed.

### Radiotherapy and chemotherapy

The definitive radiotherapy included external beam radiotherapy (EBRT) to pelvic ± para-aortic fields and high-dose rate brachytherapy (HDR-BT). EBRT were carried out by three-dimension conformal radiotherapy (3D-CRT) or Intensity modulated radiotherapy (IMRT), the radiation boost to LNs were completed by IMRT.

Fractionation, dose, field, and technique of radiation were recorded. EBRT was administered daily, 5 consecutive days per week, with a total dose of 45–50.4 Gy in 25–28 fractions with energy of 6 or 10 MV photons. The radiation dose escalation to LNs were delivered by simultaneous integrated boost (SIB) or sequential boost (SEB) with a total dose of 50–74.4 Gy (2.0–2.2 Gy/fraction). Dose escalation to 60–74.4 Gy was performed when the lymph node still larger than 1 cm or marked enhancement of CT scan after receiving dose of 50–55 Gy. The total dose to the lymph nodes was decided according to the clinical protocol in the treating center, including the size, location, adjacent organs, radiation sensitivity of LNs and the general condition of patients.

In total, 708 (70.7%) patients received CCRT. In the present study, 2–6 cycles (median, 5 cycles) of weekly cisplatin (40 mg/m^2^) or 2 cycles every 3 weeks of cisplatin (60 mg/m^2^) and 5-fluorouracil (1000 mg/m^2^) or 2 cycles every 3 weeks of cisplatin (60 mg/m^2^) and paclitaxel (150 mg/m^2^) were administered. After completion of CCRT, 368 (36.7%) patients received additional 2–4 cycles of consolidation chemotherapy using cisplatin and 5-fluorouracil or cisplatin and paclitaxel. Most patients received weekly cisplatin CCRT, combined regimen CCRT and consolidation chemotherapy were considered only when patients were judged to have a poor prognosis (bulky cervical tumor, bulky LNs, positive para-aortic LNs, extensive LN metastasis).

### Evaluation of LNs

Magnetic resonance imaging (MRI) or computed tomography (CT) were routinely used to indicate whether patients have metastatic LNs. 18.8% (188/1002) of patients underwent additional ^18^F-fluorodeoxyglucose (FDG)-positron emission tomography (PET)/CT scan. The imaging data of all patients were reviewed and the short-axis diameter of all LNs detected in pelvic and para-aortic areas were recorded for analysis. The clinical criterion for a metastatic LN was a short axis of LN ≥ 1 cm or increased FDG uptake (≥ 2.5) in the areas irrelevant to physiologic or benign sites on PET [19, 20]. Some LNs of short axis < 1 cm without PET/CT scan also had dose escalation according to radiologist clinical experience. Therefore, LNs < 1 cm were included to evaluate the value of dose escalation for those patients. For patients with multiple LNs, the largest LN size was used for grouping during the survival analysis.

### Follow-up

After completion of treatment, patients had follow-up visits every 3 months during the first 2 years, every 6 months during 2–5 years, and once a year thereafter. The examinations consisted of gynecological examination, blood tumor markers, ultrasonography, MRI, and CT scan. LN control was defined as patients without progression of LNs and no new development of pelvic and para-aortic nodal metastasis within EBRT field in follow-up MRI or CT scan during the first year.

Control and survival data were captured. Toxicities in gastrointestinal and hematopoietic systems were reviewed from the clinical data, while they were scored according to the toxicity criteria of Radiation Therapy Oncology Group (RTOG) [21]. The median follow-up time was 79 months.

### Statistical analysis

Continuous variables were expressed as mean ± standard deviation or median (min–max), and categorical variables were presented as number/percentage. The t-test was used to compare quantitative data, while the Chi-square test or the Fisher’s exact test was used for comparing qualitative data (two-sided tests). Progression-free survival (PFS) and overall survival (OS) were defined as time in months from the start of radiotherapy to the date of event or the last follow-up visit. Survival probability in different groups was calculated using the Kaplan–Meier method with the log-rank test. The receiver operating characteristic (ROC) curve analysis was employed to determine optimal cut-offs for radiation dose that would predict good control of LNs. P < 0.05 was considered statistically significant. Statistical analyses were performed using SPSSv.26 (IBM Corporation, Armonk, NY, USA).

## Results

### Patients’ characteristics

A total of 1002 patients were involved in our study. Patients’ characteristics are presented in Table 1. There were 77 (7.7%) patients in stage IB1–IIA2, 456 (45.5%) patients in stage IIB, 62 (6.2%) patients in stage IIIA, 393 (39.2%) patients in stage IIIB, and 14 (1.4%) patients in stage IVA. Patients’ median age was 56 (range 25–90) years old. The histopathological types included squamous cell carcinoma (n = 970, 96.8%), adenocarcinoma (n = 29, 2.9%), and adenosquamous carcinoma (n = 3, 0.3%). Besides, 580 (57.9%) patients were detected with 1310 LNs, 491 (49%) patients were detected with pelvic LNs only, and 89 (8.9%) patients were detected with para-aortic LNs with or without pelvic LNs. The median number of LNs per patient was 2 (rang, 1–16).

**Table 1:** Patients’ characteristics

Characteristics	n (%)
Age, years	
Median	56
Range	25–90
FIGO stage	
IB1–IIA2	77 (7.7)
IIB	456 (45.5)
IIIA	62 (6.2)
IIIB	393 (39.2)
IVA	14 (1.4)
Histology	
Squamous carcinoma	970 (96.8)
Adenocarcinoma	29 (2.9)
Adenosquamous carcinoma	3 (0.3)
Concurrent chemotherapy	
Yes	708 (70.7)
No	294 (29.3
Consolidate chemotherapy	
Yes	368 (36.7)
No	634 (63.3)
Three-dimensional brachytherapy	
Yes	32 (3.2)
No	
Patients with LNs	970 (96.8)
Yes	580 (57.9)
No	422 (42.1)
Patients with LNs in different locations	
Pelvic only	491 (49)
Para-aortic ± pelvic	89 (8.9)
Number of LNs	1310
Number of LNs per patient	
Median	2
Range	1–16
Short diameter of LNs	
Range, cm	0.4–4.8
< 1 cm	878 (67)
1–2 cm	374 (28.5)
≥ 2 cm	58 (4.4)
Range of LNs ≥ 2 cm, cm	2–4.8
Median of LNs ≥ 2 cm, cm	2.25

Among 1310 LNs, there were 878 (67%) nodes with short diameter < 1 cm, 374 (28.5%) nodes with short diameter ≥ 1 and < 2 cm (range 1–2 cm), and 58 (4.4%) nodes with short diameter ≥ 2 cm. The radiation doses for LNs are listed in Table 2. A total of 897 (68.5%) nodes received dose escalation, and 194 (14.8%) nodes received dose > 60 Gy. Among LNs with short diameter < 1 cm, 544 (61.9%) nodes had dose escalation and 124 (14.1%) nodes received dose > 60 Gy. Among LNs with short diameter of 1–2 cm, 301 (80.5%) nodes received dose escalation and 59 (15.8%) nodes received dose > 60 Gy. Among LNs with short diameter ≥ 2 cm, 52 (89.7%) nodes received dose escalation, and 11 (19%) nodes received dose > 60 Gy. The average dose of LNs with short diameter ≥ 2 cm (56.8 ± 5.5 Gy) was significantly higher than that of LNs with short diameter of 1–2 cm (55.2 ± 5.6 Gy) (P = 0.046) and that of LNs with short diameter < 1 cm (53.2 ± 6.2 Gy) (P = 0.000).

**Table 2:** Radiation dose of LNs

Radiation dose of LNs	n (%)	P value
All LNs	1310	
Average dose, Gy	53.9 ± 6.1	
Dose range, Gy	45–74.4	
≤ 50.4 Gy	413 (31.5)	
50.4–60 Gy	703 (53.7)	
> 60 Gy	194 (14.8)	
LNs < 1 cm	878	
Average dose, Gy	53.2 ± 6.2	
Dose range, Gy	45–69	
≤ 50.4 Gy	334 (38.1)	
50.4–60 Gy	420 (47.8)	
> 60 Gy	124 (14.1)	
LNs 1–2 cm	374	
Average dose, Gy	55.2 ± 5.6	0.000*
Dose range, Gy	45–69	
≤ 50.4 Gy	73 (19.5)	
50.4–60 Gy	242 (64.7)	
> 60 Gy	59 (15.8)	
LNs ≥ 2 cm	58	
Average dose, Gy	56.8 ± 5.5	0.000*, **0.046#**
Dose range, Gy	45–74.4	
≤ 50.4 Gy	6 (10.3)	
50.4–60 Gy	41 (70.7)	
> 60 Gy	11 (19)	

Bold values shown statistical differences (*P* < 0.05)

^*^Compared with the average dose of LNs < 1 cm

^#^Compared with the average dose of LNs 1–2 cm

### Control rates of LNs

**Fig. 1:**
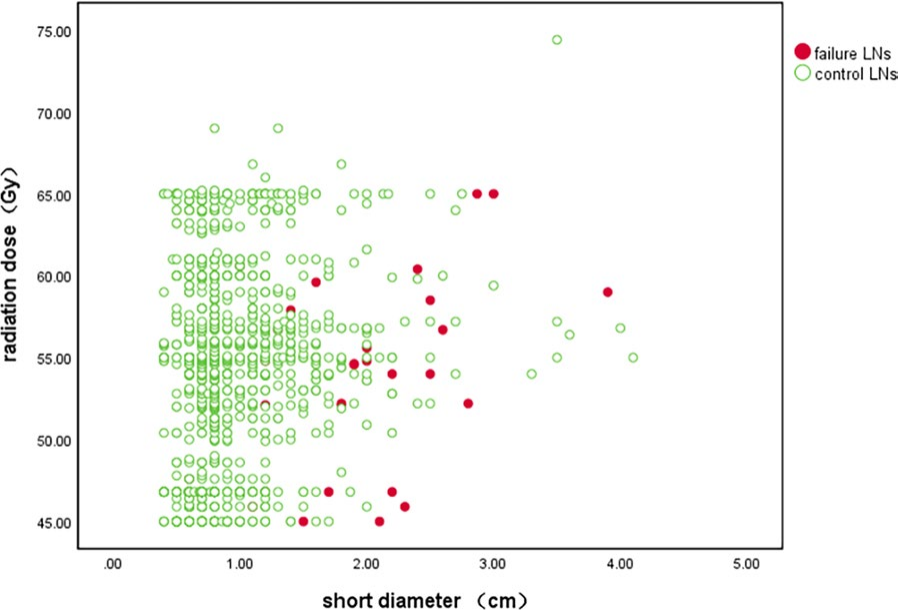
Scatter plot of LNs with different size, radiation dose and control status

The control rates of all LNs are summarized in Table 3. Besides, 1276 (97.4%) of 1310 LNs were controlled, 873 (99.4%) of 878 LNs with short diameter < 1 cm were controlled, 359 (96%) of 374 LNs with short diameter of 1–2 cm were controlled, and 44 (75.9%) of 58 LNs with short diameter ≥ 2 cm were controlled. There were significant differences among the three groups (P = 0.000). A scatter plot with the short diameter of the LNs on the horizontal axis, the dose to each node on the vertical axis and marking control status was shown in Fig. 1.

**Table 3:** Control rates of LNs

LNs	Total	Controlled, n (%)	P value
All LNs	1310	1276 (97.4%)	
LNs < 1 cm	878	873 (99.4%)	**0.000**
LNs 1–2 cm	374	359 (96%)	
LNs ≥ 2 cm	58	44 (75.9%)	
All LNs	1310	1276 (97.4%)	
≤ 50.4 Gy	413	403 (97.6%)	0.257
50.4–60 Gy	703	681 (96.9%)	
> 60 Gy	194	192 (99%)	
LNs < 1 cm	878	873 (99.4%)	
≤ 50.4 Gy	334	332 (99.4%)	0.647
50.4–60 Gy	420	417 (99.3%)	
> 60 Gy	124	124 (100%)	
LNs 1–2 cm	374	359 (96%)	
≤ 50.4 Gy	73	68 (93.2%)	0.135
50.4–60 Gy	242	232 (95.9%)	
> 60 Gy	59	59 (100%)	
LNs 1–2 cm			
< 55 Gy	171	160 (93.6%)	**0.028**
≥ 55 Gy	203	199 (98%)	
LNs 1–2 cm			
≥ 55 Gy and ≤ 60 Gy	122	119 (97.5%)	1.000
> 60 Gy	81	80 (98.8%)	
LNs ≥ 2 cm	58	44 (75.9%)	
≤ 50.4 Gy	6	3 (50%)	0.299
50.4–60 Gy	41	32 (78%)	
> 60 Gy	11	9 (81.8%)	
LNs ≥ 2 cm			
< 55 Gy	19	12 (63.2%)	0.107
≥ 55 Gy	39	32 (82.1%)	
LNs ≥ 2 cm			
≥ 55 Gy and ≤ 60 Gy	26	22 (84.6%)	0.666
> 60 Gy	13	10 (76.9%)	

Bold values shown statistical differences (*P* < 0.05)

The LNs with different sizes were assigned into three groups: radiation dose ≤ 50.4 Gy, radiation dose > 50.4 Gy and ≤ 60 Gy (range 50.4–60 Gy), and radiation dose > 60 Gy. Among all the nodes, there was no significant difference in nodal control rate among the three dose-based groups (P = 0.257). In the subgroup of LNs with short diameter < 1 cm, the nodal control rates in the three dose-based groups were very similar (99.4% vs. 99.3% vs. 100%, P = 0.647). However, among nodes with short diameter of 1–2 cm and nodes with short diameter ≥ 2 cm, although there was no significant difference, the control rate in different groups increased incrementally with the increase of radiation dose. Thus, the ROC curves were plotted for nodal dose to evaluate their predictive values for nodal control. The area under curve (AUC) of radiation dose for predicting good control of LNs with short diameter of 1–2 cm was 0.645 (95% confidence interval [CI], 0.507–0.783). The optimal cutoff value was 54.8 Gy (sensitivity of 56.0%, specificity of 73.3%, Youden's index of 0.293). Among LNs with short diameter ≥ 2 cm, the AUC was 0.582 (95% CI 0.394–0.768) and the cutoff value for radiation dose was 54.9 Gy (sensitivity of 72.7%, specificity of 50%, Youden's index of 0.227).

Thus, we assumed that the optimal target dose was 55 Gy. The control rate of LNs (1–2 cm) was significantly higher in the group of dose ≥ 55 Gy than that in group of dose < 55 Gy, as shown in Table 3 (98% vs. 93.6%, P = 0.028). The control rate of LNs (1–2 cm) in group of dose > 60 Gy was not significantly higher than that in group of doses 55–60 Gy (98.8% vs. 97.5%, P = 1.000). The control rate of LNs with short diameter ≥ 2 cm was higher in group of dose ≥ 55 Gy than that in group of dose < 55 Gy, while there was no significant difference (82.1% vs. 63.2%, P = 0.107). Besides, the control rate of LNs with short diameter ≥ 2 cm in group of dose > 60 Gy was not higher than that in group of doses 55–60 Gy (76.9% vs. 84.6%%, P = 0.666).

### Patients’ survival

**Fig. 2:**
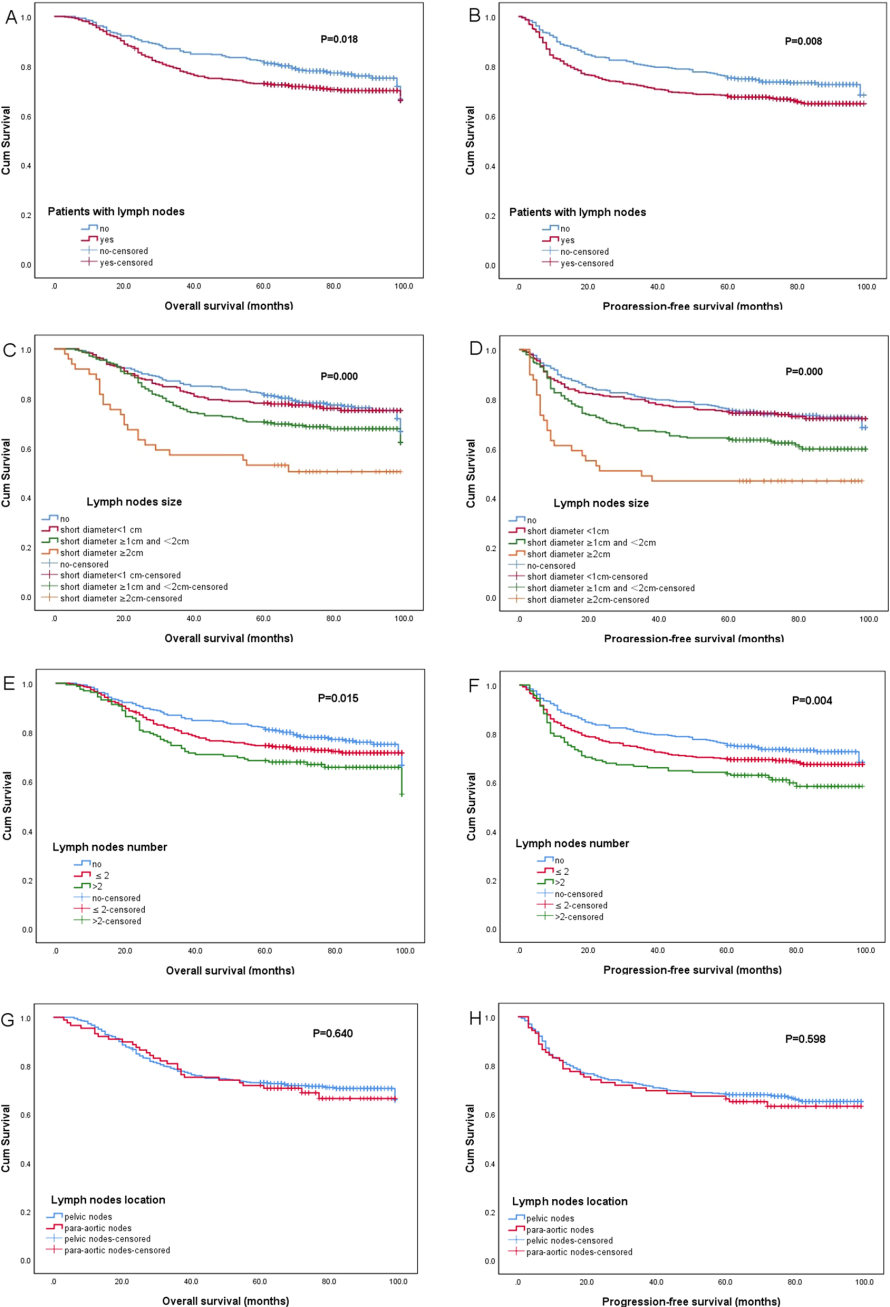
Survival curve of patients with different LN status. Overall survival (**A**) and progression-free survival (**B**) of patients with and without lymph nodes. Overall survival (**C**) and progression-free survival (**D**) of patients with lymph nodes of different sizes. Overall survival (**E**) and progression-free survival (**F**) of patients with lymph nodes of different number. Overall survival (**G**) and progression-free survival (**H**) of patients with lymph nodes of different location

Patients’ OS and PFS rates are presented in Fig. 2. The OS and PFS rates of patients with LNs were significantly lower than those of patients without LNs (5-year OS, 72.9% vs. 81.4%, P = 0.018) (5-year PFS, 67.9% vs. 75.3%, P = 0.009). Among patients with LNs with different sizes, the OS and PFS rates of patients with LNs ≥ 2 cm were significantly lower than those of patients with LNs of 1–2 cm and with LNs < 1 cm (5-year OS, 53.1% vs. 70.5% vs. 78.1%, P = 0.000) (5-year PFS, 46.9% vs. 63.9% vs. 74.7%, P = 0.000). Among patients with LNs of different number, the OS and PFS rates of patients with more than 2 LNs were significantly lower than those patients with ≤ 2 LNs and without LN (5-year OS, 68.5% vs. 74.6% vs. 81.4%, P = 0.015) (5-year PFS, 63.6% vs. 69.6% vs. 75.3%, P = 0.004). Among patients with LNs of different location, there was no significant difference in OS and PFS rates among the pelvic and para-aortic (with or without pelvic nodes) groups (5-year OS, 73.1% vs. 71.9% P = 0.640) (5-year PFS, 68.2% vs. 66.3% P = 0.598).

**Fig. 3:**
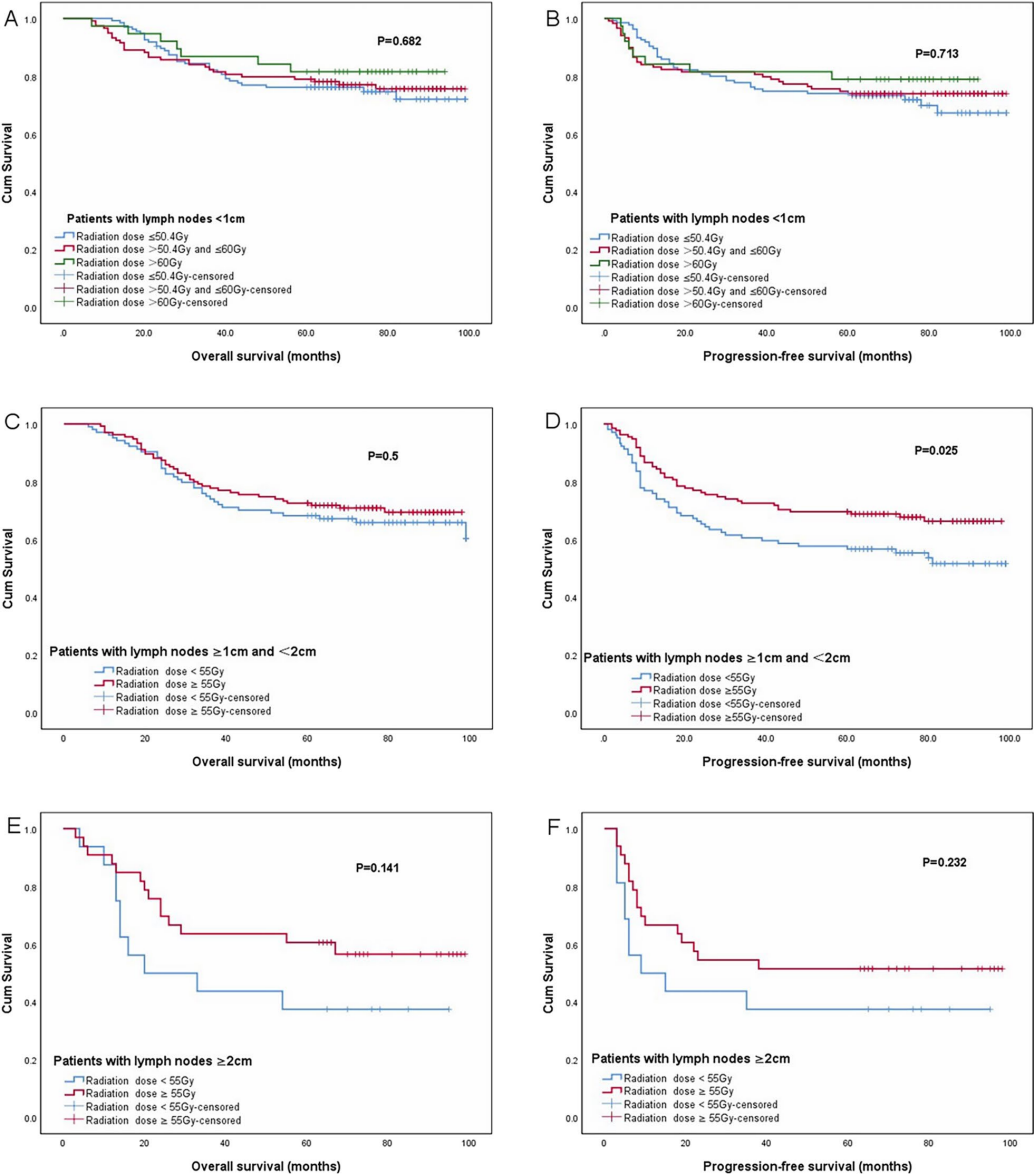
Survival curves of patients with different dose escalation of LNs. Overall survival (**A**) and progression-free survival (**B**) of patients with lymph nodes < 1 cm received different radiation doses. Overall survival (**C**) and progression-free survival (**D**) of patients with lymph nodes of 1–2 cm received different radiation doses. Overall survival (**E**) and progression-free survival (**F**) of patients with lymph nodes ≥ 2 cm received different radiation doses

In the subgroup analysis of LNs with different sizes, the OS and PFS rates of patients with different escalated doses are shown in Fig. 3. Among patients with LNs < 1 cm, there was no significant difference in OS and PFS rates among the three dose-based groups (≤ 50.4 Gy, 50.4–60 Gy, and > 60 Gy) (5-year OS, 76.2% vs. 79% vs. 81.6%, P = 0.682) (5-year PFS, 74.1% vs. 73.9% vs. 78.9% P = 0.713). Among patients with LNs of 1–2 cm, the 5-year PFS rate was significantly higher in group of dose ≥ 55 Gy than that of dose < 55 Gy (69.6% vs. 56.7%, P = 0.025). However, there was no difference in 5-year OS rate of the two dose-based groups (72.6% vs. 68.3%, P = 0.5). Among patients with LNs ≥ 2 cm, the 5-year OS and PFS rates were higher in group of dose ≥ 55 Gy than those in group of dose < 55 Gy, while there was no significant difference between the two groups (5-year OS, 60.6% vs. 37.5%, P = 0.141) (5-year PFS, 51.5% vs.37.5%, P = 0.232).

### Patterns of nodal recurrence

Overall, 90 patients (9.0%) developed nodal recurrence with 22 patients having pelvic, 42 para-aortic, 27 supraclavicular, 5 inguinal, 4 mediastinal, and 2 axillary nodal recurrence, accounting for 24.4%, 46.6%, 30%,5.6%, 4.4%, and 2.2% of all patients with nodal recurrence, respectively (Table 4). Nodal recurrence in a single nodal region was reported in 76 (84.4%) patients and in multiple regions was reported in 24 (26.7%) patients. Single nodal region recurrence was reported in 21.1% of patients with pelvic recurrence, 36.7% of patients with para-aortic recurrence and 21.1% of patients with supraclavicular recurrence. Of the patients with nodal recurrence, 68.9% were located outside the elective target, 27.8% inside the target.

**Table 4:** Patterns of nodal recurrence

Patients	n (%)
All patients	1310
Patients with nodal recurrence	90 (9.0%)
Pelvic	22 (24.4%)
Para-aortic	42 (46.6%)
Supraclavicular	27 (30%)
Inguinal	5 (5.6%)
Mediastinal	4 (4.4%)
Axillary	2 (2.2%)
Patients with nodal recurrence	90
Single region	76 (84.4%)
Pelvic	19 (21.1%)
Para-aortic	33 (36.7%)
Supraclavicular	19 (21.1%)
Inguinal	4 (4.4%)
Axillary	1 (1.1%)
Multiple regions	24 (26.7%)
Patients with nodal recurrence	90
Inside the target	25 (27.8%)
Outside the target	62 (68.9%)
Inside and outside target	3 (3.3%)

### Complications

According to the toxicity criteria of RTOG, complications with acute radiation morbidity grades 3 and 4 are listed in Table 5. Among the three dose-based groups of ≤ 50.4 Gy, 50.4–60 Gy, and > 60 Gy, the acute radiation morbidity grades 3 and 4 for hematologic white blood cell (WBC), neutrophils, and hemoglobin were significantly different and increased with the elevation of dose (Hematologic WBC, 37.1% vs. 48.5% vs 60.8%, P = 0.000) (Neutrophils, 27.5% vs. 36.2% vs 39.2%, P = 0.006) (Hemoglobin, 13.4% vs. 22.9% vs 24.7%, P = 0.000). However, there was no significant difference among the acute radiation morbidity grades 3 and 4 for platelets, lower gastrointestinal (GI), and upper GI (Platelets, 6.4% vs. 9.2% vs. 9.3%, P = 0.245) (Lower GI, 15% vs. 10.9% vs. 9.3%, P = 0.111) (Upper GI, 20.6% vs. 25.9% vs. 16.5%, P = 0.079).

**Table 5:** Acute radiation toxicity (RTOG criteria)

Characteristics	Total	Grade 3 and 4 case, n (%)	P value
Hematologic WBC			
≤ 50 Gy	612	227 (37.1)	**0.000**
50.4–60 Gy	293	142 (48.5)	
> 60 Gy	97	59 (60.8)	
Neutrophils			
≤ 50 Gy	612	168 (27.5)	**0.006**
50.4–60 Gy	293	106 (36.2)	
> 60 Gy	97	38 (39.2)	
Hemoglobin			
≤ 50 Gy	612	82 (13.4)	**0.000**
50.4–60 Gy	293	67 (22.9)	
> 60 Gy	97	24 (24.7)	
Platelets			
≤ 50 Gy	612	39 (6.4)	0.245
50.4–60 Gy	293	27 (9.2)	
> 60 Gy	97	9 (9.3)	
Lower GI			
≤ 50 Gy	612	92 (15)	0.111
50.4–60 Gy	293	32 (10.9)	
> 60 Gy	97	9 (9.3)	
Upper GI			
≤ 50 Gy	612	126 (20.6)	0.079
50.4–60 Gy	293	76 (25.9)	
> 60 Gy	97	16 (16.5)	

Bold values shown statistical differences (*P* < 0.05)

## Discussion

Patients with cervical cancer and positive LNs had significantly poorer survival outcomes [5–8]. In our study, the OS and PFS rates of patients with LNs were significantly lower than those of patients without LN. And we found the size and the number of LNs were important prognostic factors of survival, which were in line with those reported previously [10, 16, 17]. To improve this condition, recent studies have recommended that radiation dose escalation for positive LNs can improve the control rate [10, 17, 22–24]. However, whether dose escalation actual improve control and survival outcomes has not been confirmed. The optimal dose required to control LNs still remains controversial, especially for nodes with different sizes.

To answer this question, we separately analyzed patients with LNs with different sizes. At present, most of the studies on dose escalation for LNs have concentrated on patients with LNs ≥ 1 cm, while dose escalation in patients with LNs < 1 cm was also administered in clinical practice. In Grigsby et al.’s study, LNs < 1 cm received 66.8 Gy, 0/76 failure in PET negative nodes; 3/89 failures in PET positive nodes [24]. Other studies showed that patients with relatively small LNs (< 1 cm) did not benefit from dose escalation [16, 17]. In our study, among the LNs (< 1 cm), 61.9% of LNs received dose escalation. We found that the dose escalation did not improve the nodal control rate and survival outcome, and the control rate (99.4%) was satisfactory without dose boost. During this period in our center, only 44 patients with LNs < 1 cm received PET/CT scan. The number of these patients was not adequate to perform subgroup analysis for the evaluation of dose escalation in LNs with or without increased FDG uptake. However, our study Included 878 LNs < 1 cm, and the nodal control rate with or without escalation was more than 99%. The PFS and OS rates of patients with LNs < 1 cm were similar with those patients without LN, and were similar with each other in different dose-based groups. Therefore, we concluded that LNs with the short diameter < 1 cm did not need radiation dose escalation in the definitive radiotherapy of cervical cancer, even if some small LNs harbor microscopic cancer cells.

To date, several studies demonstrated that the dose escalation of LNs could improve the control rate of patients with LNs ≥ 1 cm [7, 9, 12–14]. However, the radiation dose greatly varied among different centers. There were two retrospective studies, which reported that radiation dose > 50.4 Gy could fully control LNs [17, 22]. Vargo et al. showed a control rate of 94% with nodal doses of 55 Gy given in 25 fractions [25]. Wakatsuki et al. suggested that nodal dose escalation > 58 Gy might be required to optimize nodal control, while Bacorro et al. recommended that dose escalation threshold should be 57.5 Gy to control LNs [9, 15]. Hata et al. demonstrated that dose boost depending on nodal size, 50.4 Gy for LNs < 2.4 cm and 55.8 Gy for LNs ≥ 3 cm, could control LNs as high as 98% [12]. Those researches on dose escalation were all retrospective studies with small sample sizes, and no consensus has reached.

To find out the dose escalation threshold for controlling LNs ≥ 1 cm, we plotted the ROC curves for node dose to evaluate their predictive values for nodal control. In patients with LNs of 1–2 cm, we found that dose ≥ 55 Gy could significantly improve the control and PFS rates, but escalating the dose to 60 Gy did not further improve the control rate. Therefore, the dose of 55 Gy would be enough for controlling 98% of LNs of 1–2 cm. In patients with LNs ≥ 2 cm, the nodal control, OS and PFS rates in the group of dose ≥ 55 Gy were higher than those in group of dose < 55 Gy, while there was no significant difference, and the control rate was not satisfactory (82.1%) in the group of dose ≥ 55 Gy. Escalating the dose higher than 55 Gy maybe required to improve the control rate. The number of LNs ≥ 2 cm in our study was relatively small, and further studies with larger sample sizes should be conducted.

The pattern of nodal recurrence in patients with cervical cancer treated with definitive radiotherapy was described in the study. The overall number of patients developing nodal failure was low (9%), which was in line with the description of the EMBRACE study [26]. Nodal recurrences were more often seen in the para-aortic region (46.6%), the second was supraclavicular region (30%). About 68.9% of all recurrences were reported outside the treatment targets, and the recurrence rate was low inside the target (27.8%).

Although IMRT facilitates the dose escalation and limits radiation dose to normal tissues, there are still some concerns regarding the possibility of increasing toxicities [23, 25]. Gogineni et al. found that acute grade ≥ 2 GI toxicity was not associated with radiation dose > 50.4 Gy [17]. It was similar with our results, in which we found that the dose escalation was not associated with a greater GI toxicity. However, in our study, it was revealed that the dose escalation was associated with a greater hematological toxicity, but toxicities were acceptable.

There were some limitations in our study. First, it was a retrospective study performed at a single center. Second, some studies reported that the dose of brachytherapy could contribute to pelvic LNs [27, 28], while we did not consider the dose contribution of brachytherapy to LNs because only 3.2% patients performed three-dimensional brachytherapy in the study. Third, there were only few patients who received PET/CT scan for pretreatment. Fourth, we didn’t evaluate the late toxicity of dose escalation. Despite these limitations, the large number of patients and a long-term follow-up are valuable for achievement of conclusive results in this study. The present study has important implications for the recommendation of individualized nodal radiation dose escalation for patients with cervical cancer.

## Conclusions

In summary, the size of LNs was noted as an important negative prognostic factor of survival in the definitive radiotherapy of patients with cervical cancer. Individualized dose recommendation is necessary for patients with LNs of different sizes. Radiation dose escalation is unnecessary for patients with LNs < 1 cm. For patients with LNs of 1–2 cm, dose escalation ≥ 55 Gy was found enough to improve the control rate and PFS rate. For patients with LNs ≥ 2 cm, dose escalation showed to improve the nodal control and survival, while further studies with larger sample sizes are essential.

## Data Availability

The datasets used and analyzed during the current study are available from the corresponding author on reasonable request.

## Abbreviations

LN: Lymph node
FIGO: International Federation of Gynecology and Obstetrics
OS: Overall survival
PFS: Progression-free survival
CCRT: Concurrent chemoradiotherapy
EBRR: External beam radiotherapy
HDR-BT: High-dose rate brachytherapy
3D-CRT: Three-dimension conformal radiotherapy
IMRT: Intensity modulated radiotherapy
SIB: Simultaneous integrated boost
SEB: Sequential boost
MRI: Magnetic resonance imaging
CT: Computed tomography
PET: 18F-fluorodeoxyglucose-positron emission tomography
FDG: 18F-fluorodeoxyglucose
RTOG: Radiation Therapy Oncology Group
ROC: Receiver operating characteristic
CI: Confidence interval
AUC: Area under curve
